# “After the Dust Settles”: Foucauldian Narratives of Retired Athletes' “Re-orientation” to Exercise

**DOI:** 10.3389/fspor.2022.901308

**Published:** 2022-07-08

**Authors:** Luke Jones, Zoe Avner, Jim Denison

**Affiliations:** ^1^Department of Sport Health and Exercise Science, University of Hull, Hull, United Kingdom; ^2^Sport, Exercise and Rehabilitation, Northumbria University, Newcastle upon Tyne, United Kingdom; ^3^Faculty of Kinesiology, Sport, and Recreation, University of Alberta, Edmonton, AB, Canada

**Keywords:** Foucault, sports retirement, exercise, movement, ethical movement practices

## Abstract

One aspect of sports retirement that has been overlooked until recently is the manner in which retired athletes relate to, and seek to redefine, the meaning of exercise in their post-sport lives. In this article, three Foucauldian scholars present and analyze a series of vignettes concerning their own sense-making and meaning-making about exercise following their long-term involvement in high-performance soccer (authors one and two) and distance running (author three). In doing so, this paper aims to underline the problematic legacy of high-performance sport for retiring athletes' relationship to movement and exercise, and to highlight how social theory, and Foucauldian theorization in particular, can serve to open new spaces and possibilities for thinking about sports retirement.

## Introduction

Living one's life after leaving high performance sport has always been an experience laced with difficulty (Mihovilovic, 1968; Hill and Lowe, 1974). Despite decades of research into this phenomenon, challenging experiences continue to be reported for those managing their lives “post-sport” (see the systematic reviews of Park et al., 2013; Fuller, 2014; Barth et al., 2020). For instance, studies from across the globe point to repeated examples of transitional challenges amongst former elite level Association football (soccer) players (Curran, 2015; Stamp et al., 2021), as well as collegiate (Barcza-Renner et al., 2020), and Olympic level track and field athletes (Ungerleider, 1997). Common problems reported by retired athletes include but are not limited to: difficulty forming alternative identities away from sport (Carless and Douglas, 2009), depression, suicide, substance abuse, and disordered eating (see systematic review by Fuller, 2014).

While the challenges associated with retirement from high-performance sport have been extensively researched, albeit dominantly through a psychological or psycho-social lens (e.g., Lally, 2007; Warriner and Lavallee, 2008; Martin et al., 2014), one aspect of sports retirement that has been overlooked until recently is the manner in which retired athletes relate to, and seek to redefine, the meaning of exercise in their post-sport lives (Tracey and Elcombe, 2004; Jones and Denison, 2019). In the current paper, we attend to this lacuna by drawing on a Foucauldian lens to present and analyze a series of vignettes from the three authors concerning their own sense-making and meaning-making about exercise following their long-term involvement in high-performance soccer (authors one and two) and distance running (author three). For us, Foucault's conception of discipline aligns strongly with a number of effects and consequences that help to understand retired athletes' experiences from long standing involvement in organized sport. More specifically, we aim to discuss how our development as Foucauldian scholars has enabled us to challenge and reformulate our relationship with exercise as retired sportspeople. Associated with this aim, importantly, are a number of larger implications for the conduct of future sport retirement research that we also intend to discuss, for example, the need to shift the dominant way of thinking about this phenomenon that is currently overwhelmingly focused upon fixing or healing the athlete in the immediate aftermath of their initial retirement transition to consider the various social relations in play that impact retired athletes' experiences.

In this paper, we define “high-performance sport” as a full-time competitive bodily endeavor, typified by the representation of a community group/institution and compensated by finance (including scholarships) and/or status, access, and affiliation. In what follows, we elaborate on how Foucauldian theorizing has been applied to date to examine high-performance sport as a modern discipline. We do so to explain what this theorizing can tell us about the lasting effects of a career in high-performance sport. Specifically, we do so by concentrating upon one aspect of a retired athlete's experience, that of their latter sport and/or exercise participation and relationship to movement. We then outline our research process followed by the presentation and analysis of our vignettes of post-sport exercise experiences.

## High-Performance Sport: The Politics of Docility Amidst a Destructive “Modern Discipline”

In his seminal work *Discipline and Punish*, Foucault (1995) outlined the specific techniques and instruments through which the body became both the object and target of power in the transition to modern society. The workings of this integrated modern system of control, known as “anatomo-politics” and its various problematic effects have been extensively mapped in the sociology of sport (see for instance, Heikkala, 1993; Rail and Harvey, 1995; Shogan, 1999; Markula and Pringle, 2006; Gearity and Mills, 2012). In particular, Shogan (1999) was amongst the first scholars to comprehensively outline how disciplinary power operates in high-performance sport through various disciplinary techniques related to the control of time, space and activity. As Shogan further argued, most modern high-performance sporting contexts can be read as “ideal” applications of modern disciplinary power, not least given the apparent productivity that appears to follow when sport is coached as a modernist foundation (Denison et al., 2013). Given this apparent productivity, many continue to argue that high-performance sports contexts act as a forum for athletes to display brilliance, find belonging and meaning, and act as role models for future generations. What is more, the role of sport as a forum for learning worthwhile life skills and for the positive psycho-social development of youth has been discussed at length (Camiré et al., 2012; Holt et al., 2020). Sports participation then, is consistently articulated as being “beneficial for the overall physical and mental health of youth” (Varghese et al., 2022, p. 20). Therefore, while we proceed to critique aspects of the high-performance sport arrangement, we also concede that a significant body of research has posited clear benefits of “well-managed sport” for society as well as individuals (see Edwards and Rowe, 2019). Accordingly, sport remains incredibly popular worldwide and is viewed as a positive social phenomenon by many adherents to what Coakley (2015) has called the “Great Sport Myth,” where sport's instrumental capacity is broadly celebrated and promoted.

Despite the above claims regarding the productivity and instrumental benefits of sport for society and individuals, Foucauldian studies (in drawing attention to the role of high-performance sport as a “modern discipline”), have highlighted various problematic effects related to the uncritical application of disciplinary techniques and instruments therein. These include; underperformance, athletic disinvestment, body-image disorders, depression and mental health issues and the celebrated production of overly compliant docile bodies (e.g., Johns and Johns, 2000; Gearity and Mills, 2012; Denison et al., 2017). Indeed, the disciplinary logic that underpins the production of elite athletes is one that seeks to shape a very particular type of athlete—namely a docile athlete that conforms to the norms and expectations of their lived space. Unsurprisingly therefore, high-performance sport can also be experienced as a coercive space where docile bodies are fabricated to fit into the expected hierarchy and social order (Markula and Pringle, 2006). High-performance sport has also been characterized as a physically dangerous, psychologically damaging, toxic and destructive hypercompetitive sub-culture where cultural capital is accrued by those who are willing to risk their ethics, morals, and health and wellbeing for better performance (Feddersen et al., 2020). What is more, being a high-performance athlete often requires adhering to normalized work expectations which are hazardous to the mental and physical health of athletes (Johns and Johns, 2000; Cushion and Jones, 2006; Roderick, 2006; Barker-Ruchti and Tinning, 2010; Gerdin et al., 2019; Jowett et al., 2020), and where athletes' bodies are routinely and ceaselessly monitored (Manley and Williams, 2022). This has sadly never been more clearly evidenced than in recent high-profile accounts of athlete abuse in the global media such as the abuse of female US gymnasts documented in the 2020 movie *Athlete A*, or the well-publicized culture of intimidation from within the British cycling team that became front page news in 2017.

Unfortunately, despite the problematic consequences highlighted thus far, the connection between discipline and the production of athletes as an “effective” approach remains strong within most high-performance sporting contexts (Denison et al., 2013). Consequently, the procurement of docility is an unfolding that, given its apparent surface benefits, e.g., winning and performance, is prioritized in high-performance sports settings (Heikkala, 1993). This prioritization occurs within the coaching community, where practitioners see no need to deviate from a range of “normal” practices centered around surveillance and control (Denison et al., 2019). As a result, the “journey” of how the coach facilitates an individual or group's docility goes largely unquestioned. Rather, it is often glamorized in popular characterizations of sports subcultures, for example in the romanticized depiction of coaches who successfully employ strategies steeped in authoritarian coaching styles (Jones, 2020).

To begin to understand how and why the currency behind the development of athletes in this manner has not been destabilized (despite its clear limitations), at this juncture, it is necessary to acknowledge and explore the complexity of docility for athletes. For example, Foucault's (1995) understanding dictates that while the disciplinary techniques and instruments that are abundant in high-performance sports settings are powerful, they must not be considered as totalizing, or as producing “complete” docility. Foucault was quite clear that individuals are not to be thought of as passive dupes with no agency. His productive articulation of power relations implied opportunities for resistance and for the negotiation and alteration of dominant discourses and power relations (Markula and Pringle, 2006). As he infamously stated, “where there is power, there is resistance, and yet, or rather consequently, this resistance is never in a position of exteriority in relation to power” (Foucault, 1978, p. 95–96).

We are fully aware that despite the intense structures that define their existence, athletes are always shaping and interacting with the sports space and context they inhabit (Clark and Markula, 2017). Nonetheless, it is clear that the settings athletes occupy are routinely choreographed to elicit compliance and to position athletic bodies as “machines” to be manipulated, enhanced and transformed (Mills and Denison, 2018). However, Foucault's theoretical schema dictates that we must also acknowledge the simultaneously relational and productive aspects of power (Clark and Markula, 2017). Moreover, in certain conditions (even those as constraining as high-performance sport) each individual will respond to disciplinary power in their own complex way (including over the long and the short-term). Along these lines, we acknowledge the importance of a healthy debate surrounding the positives to be taken from a life in sport and the extent to which athletes can influence their own fortunes and identities during (Roderick, 2014) and after a sporting career (Carless and Douglas, 2009; Hickey and Roderick, 2017; Stamp et al., 2021). Research from educational psychology also indicates that individuals' dispositions develop and evolve over the course of a lifetime (Bloomer and Hodkinson, 2000), something much of the sport retirement literature has yet to acknowledge, but a facet of athletes' retirement experiences that we certainly recognize as significant.

That said, as Foucauldians, well versed in the analysis of high-performance and elite sports subcultures over a number of years, the position we take for the current paper is that, on balance, it is hard to ignore (given the overwhelming body of evidence), that immersion in high-performance and elite sport has significant and multiple long-term consequences for the individuals concerned. More precisely, that when one considers the likely and reasonable response of a docile athlete in the presence of these coercive, objectifying relations of power, the far more likely and common outcome is one of acceptance, mimicry, and/or silence (Johns and Johns, 2000), an outcome that has significant implications for both the performing and former athlete (Jones and Denison, 2017; Denison, 2019). Regardless of how messy and tangled its workings are, we purport that it is not prudent nor even realistic to seek a complete “undoing” of disciplinary power (Denison et al., 2017). Rather, (as this paper moves to demonstrate) perhaps a better strategy is, in the first instance, to attune oneself as sensitively as possible to its multiple, even paradoxical, immediate, and lasting effects.

In line with this sentiment, in what follows, we consider how our Foucauldian approach to research has helped us to engage differently with our present movement and exercise practices, having now stepped away from high-performance sport. We hope that our approach depicted here helps catalyze a new trend in sports retirement research, where instead of the archetypal focus upon the acute challenges of initial transition and identity disruption, our new heuristic frames the retirement experience, and in particular exercise therein, as a more ongoing delineated process over time. Before we move on to do this through a presentation and analysis of our own vignettes, we first provide a review of the literature on the legacy of an immersion in high-performance sports culture.

## “After the Dust Settles”: The Legacy of an Immersion in High-Performance Sports Culture

Barth et al. (2020) identified that retirement studies have predominantly considered the “initial phase” where athletes transit away from their competitive sport. In this section we review the longer-term implications for athletes and their experiences of what has been labeled by some as the “post-retirement phase” (Stambulova et al., 2020). Below, we briefly review the physiological, psychological, emotional and social implications for athletes who have spent a significant period of time immersed in the “modern discipline” (Markula and Pringle, 2006) of a high-performance sports culture.

While it is largely agreed that physical activity in the form of sport is important for physical health, research from the field of sports medicine has also established that there can be limiting longer-term implications for an individual's physical health related to having been a high-performance athlete. This includes higher levels of physical dysfunction, depression, fatigue, sleep interruption and pain (Simon and Docherty, 2014) alongside consequences of repeated concussions in sport, such as depressive episodes, chronic pain and the development of neurodegenerative diseases (e.g., Alzheimer's, Parkinson's and dementia, Lehman et al., 2012).

The psychological benefits of appropriate physical activity in the form of sport are also well recognized (Eime et al., 2013), including for an individual's mental health (Carless and Douglas, 2011). However, sport psychology research has also emphasized the significant connections between high-performance sports participation and longer-term mental health issues (Brewer et al., 1993). Importantly, recent sport psychology research has identified an even stronger relationship between sports retirement and depressive symptomatology than previously believed (van Ramele et al., 2017). According to these studies, the strength and exclusivity of the athletic role during sport participation increases athletes' potential vulnerability to psychological distress after leaving sport, especially if this attachment to athletic identity is maintained into retirement (Sanders and Stevinson, 2017). It is unsurprising therefore, that existing suggestions within sports psychology literature promote identity management during and after a sports career to be of primary importance in mitigating the dangers associated with this critical transition (Carless and Douglas, 2009).

While sport sociologists also share these concerns (Mihovilovic, 1968; Coakley, 1992), their framing of sporting transition and retirement issues and the problem-solving approaches they draw upon differ somewhat. For example, removal from a “sports work” space has been identified as extremely challenging (Curran, 2015) and, more recently, has been articulated as a social rather than isolated process (Hickey and Roderick, 2017; Stamp et al., 2021), that includes an unpredictable, paradoxical fluctuation between painful and liberating emotions (Jones and Denison, 2017).

Foucault has been used across the field of sport sociology to explore a host of issues related to the imposition of discipline (Markula and Pringle, 2006), including body image disorders (Johns and Johns, 2000; McMahon et al., 2012), poor athletic performance and disinvestment (Denison, 2007), and suicide and depressive symptomology (Gerdin et al., 2019). Within this body of literature, the implications of retirement (and in particular those over the longer term) have not received such detailed attention. Of particular note, in one example, Jones and Denison's (2017) Foucauldian informed study established some important connections between retired footballers' struggles with adjusting to life after sport and the “various arrangements of disciplinary power operating within their sporting context” (p. 937). According to these authors, the imposition of discipline during athletes' careers not only renders them “docile” during their athletic careers but also has significant implications for how a retired athlete understands and experiences their dynamic and multifaceted retirement journey. What Jones and Denison and others have therefore argued for is a shift in focus away from equipping individual transitioning athletes with coping tools and strategies toward challenging the specific discourses, power relations, and structures of high-performance sport that can lead to problematic transition and retirement experiences in the first place. Crocket's (2014) paper has similarly demonstrated that Foucault's cache of concepts are helpful to think about various aspects of retirement in new and different ways. Crocket's Foucauldian study in the context of ultimate Frisbee, also highlighted how retired athletes problematized their ongoing participation in the “truth games” of their previous sports. In doing so his participants found that they no longer wanted to adhere to these games of truth in retirement, and rather were moved to re-evaluate their relationship to sport and exercise.

To summarize, our overview of the relevant post-retirement research has emphasized the key issues that retiring athletes face. These include but are not limited to: challenges surrounding their physical and mental health, the management of their evolving identities, and the negotiation of the new social contexts they experience in their post-sport lives. This review reiterates Barth et al. (2020) recent observation that the significant majority of published papers examining retirement from elite sport have predominantly focused upon the *initial* transition into retirement (Hickey and Kelly, 2008; Jones and Denison, 2017; Stamp et al., 2021). Moreover, while a handful of examples of sports retirement research that entertain the longer-term psycho-social impact of a sports career upon retirement experience do exist (McMahon et al., 2012; Agnew and Drummond, 2015; Torregrosa et al., 2015), including the longer-term connection between an athlete's career demands and their experiences of exercise in retirement (Tracey and Elcombe, 2004; Jones and Denison, 2019), there remains a paucity of research from any tradition that considers the implications of having been an obedient and compliant (read “docile”) body, beyond the frequently analyzed initial “retirement transition” (Fuller, 2014; Barth et al., 2020).

Put simply, socio-cultural researchers who analyze the phenomenon of retirement from high-performance sport have yet to extensively consider the longer-term social implications of having “lived in sport”—after the dust has settled, as it were, due to the specific discourses and power relations present and active within high-performance sport systems and cultures. Importantly, our aim in the current paper is to take the field of sports retirement research toward developing a deeper understanding of the heritage and legacies of these longer-term implications, about which very little is known. For example, how do former high-performance athletes negotiate their complex relationships to exercise that are often interwoven with historical relationships to pain, to injury, to ways of eating and drinking formulated during a career? And, how do these individuals relate to, or attempt to develop new meanings for exercise in retirement? To address this gap in the socio-cultural research on sport retirement, we have taken it upon ourselves as both retired athletes and socio-cultural sport scholars who work from a Foucauldian perspective, to illustrate how it can be possible to resist and challenge the often problematic legacy of discipline that many retired athletes carry with them into their post high-performance sporting lives.

## The Research Process

Having considered what research has discovered about the legacy of immersion within the modern discipline of sport, we now introduce our research process. We do so to explain and legitimize our decision to present a selection of our narratives about our experiences of exercise after sport. All three contributing authors have, in the past, competed as high-performance athletes across a range of countries and cultures. The First Author competed in youth international soccer and worked within semi-professional soccer in the United Kingdom (UK) in the early part of the 21st century. He retired in 2006. The Second Author competed in youth international soccer and played semi-professionally for various clubs in France, the UK, the United States (US), and Canada. She retired in 2010. The Third Author was a NCAA Division I runner in the mid-1980's followed by a 5 year period where he competed internationally while also coaching high school and university runners in the US. He retired in 1992. Since retiring from our respective sports, we moved into academia where we trained to be, and now operate as, Foucauldian scholars of sport. We were particularly drawn to Foucault given the symmetry of his concepts with the body and performance in highly regulated settings such as high performance sport.

In this project, we have adopted an auto-ethnographic approach (Ellis and Bochner, 2000) to tell our stories. Auto-ethnography is a research method that utilizes researchers' autobiographical data to thickly describe, locate, and analyze their experiences (Chang, 2008). More precisely, we have embraced an “analytic” auto-ethnographic approach (Anderson, 2006) to each contribute a narrative “vignette” (Tsang, 2000; Rinehart, 2010) portraying our own complex and non-linear experiences of exercise after high-performance sport. Once we had all read and commented upon each other's vignettes, we then collectively analyzed them using our Foucauldian lens. An analytic auto-ethnographic approach speaks to a commitment to “develop theoretical understandings of broader social phenomena” (Anderson, 2006, p. 373) and was therefore especially well-suited to our Foucauldian driven approach to narrative research. This research project was further guided by some of the main features of analytic auto-ethnographic research which include member researcher status, critical reflexivity and positionality, and lastly a commitment to theoretical analysis and a blend of expression and theory (Anderson, 2006; Denison, 2016).

While each author was given the freedom to compose their vignettes in any way that they saw fit, before we began the process of putting pen to paper, we agreed that in our writings we should try to achieve three purposes. Firstly, to reveal how high-performance sport-imposed discipline upon us as athletes. Secondly, to show the reader how the effects of this imposition influenced us during and after our careers. And finally, to highlight how we now *attempt* (as we think and move differently with Foucault) to respond to these residual effects by exercising/moving in ways that we feel are healthier, more ethical, and more sustainable. Our vignettes focused on (a) the limitations of the social, physical and emotional legacy of high-performance sporting culture when initially participating in exercise practices and (b) the continuous push-pull movement between desires to experience and relate to one's body, others, and movement differently on the one hand and the strong pull of sporting practices underpinned by a familiar disciplinary logic on the other. In doing so, this paper argues for an understanding of change, which considers the complex, non-linear, relational, networked, and affective dimensions of sporting transition and retirement experiences. Thereby moving away from an emphasis upon an individual's actions, toward an appreciation of the relational effects of new arrangements of power that retired athletes now occupy.

Throughout these vignettes we foreground the importance of the Foucauldian concept of “problematization” as a critical tool to enable individuals to distance themselves from taken-for-granted and normalized social and sporting practices and to critically evaluate the (un)intended consequences of these practices and the constitution of problematic subjectivities (i.e., modes of being a subject) within and through specific relations of power-knowledge. For example, problematizing the disciplinary legacy of high-performance sport (MacKay and Dallaire, 2013; Crocket, 2014, 2015; Denison et al., 2017; Gerdin et al., 2019), the narrow and cyclical relationship between sport science knowledge and discipline (Mills and Denison, 2013; Konoval et al., 2019), and the production of docile bodies through taken-for-granted, normalized, and sanctioned “best” scientific coaching practices (Denison and Avner, 2011; Denison et al., 2017). In so doing, other forms of embodiments and relations to the self and others, the body and movement are made possible.

For Foucault (1982, p. 785), “problematization” was both a paramount task and a critical tool/technique to enable individuals to think and practice differently by allowing them to “liberate themselves from the “double-bind” of individualizing and totalizing modern power structures.” In this paper, we discuss how our encounter with this concept and more broadly with Foucauldian thought has allowed us to develop a deeper understanding of the limiting legacy of high-performance sport — particularly in relation to our bodies, and movement and exercise. In particular, this involved considering the formation of coaching knowledges and how these come to bear upon the athletic body via practices which seek to subject, use, transform and improve. The vignettes presented below were included because they reflect our evolving engagement with poststructuralist theorization and concurrent experimentation with new ways of moving and exercising—practices inspired by our active problematization of our respective high-performance sporting cultures.

## Thinking and Moving Differently With Foucault: Developing New Meanings for Exercise Post High-Performance Sport

It is important to note before moving forward that we recognize that the sports of association football and distance running are of course typified and framed by different socio-historical arrangements, traditions, and cultures. Despite these important differences, we do believe that there are significant commonalities to our experiences as former high-performance athletes, and that therefore, there is merit in assembling our experiences in this paper as part of the same critical conversation. In what follows, each of our narrative vignettes are presented in turn. We then move on to provide our collective Foucauldian commentary of all three and briefly discuss what we see as the key implications from our stories and our research approach, for the scholarship of sports retirement. Briefly, Author One has chosen to write about his experiences of recreational jogging after leaving competitive football, Author Two has chosen to write about her experiences of rock climbing after leaving competitive football, and Author Three has chosen to write about his experiences of cross-country skiing after the end of his distance running career.

### Author One's Vignette–Spring 2021

Looking back, I see that during my years in football, I willingly, eagerly surrendered my body to the rigors of discipline. I trusted in the promise that if I tenaciously adopted the prescribed, regimented routine, things would work out. I believed the company line that through my own graft, I could make the fragile resilient, the broken whole, and the erratic consistent. Yet, each injury or set-back diminished me. Each disappointment fed a growing, parasitical darkness within—borne from a confusion surrounding the misalignment between hope and fruition. The equation that I had been promised would balance, would not. The map I had been told to follow, had gotten me lost. I tried, but all the while I felt deep down that I was impotent to do a single thing about it. As a footballer, I felt forever exposed. Silently pleading for my body's capabilities to be acknowledged and to be validated by someone, something. To be considered worthy by an abstract, murky jury who passed judgment on my every moment. The unshakable feeling that someone was forever watching me, appraising me as I strengthened every muscle, kicked every ball, and ran every mile.

So, in 2006, at the age of 22, after 12 years of industry, I stopped trying to be a footballer. I needed to move on, but I didn't have the language, the vocabulary, to explain what had happened, indeed, what was continuing to happen to me. And I needed that. Boy, did I need that. That is why I will be forever grateful for the writings of Michel Foucault. His concepts gave me the stationary to ascribe a resonant narrative to my tribulations within football and to re-imagine how I might begin to move and exercise again, “to identify the limits that we may go beyond” (Foucault, 1984, p. 47). I can now confidently attribute the overwhelming memory I have of always being “on trial” to the ceaseless imposition of the discipline I experienced during my football years (Jones and Denison, 2017). Being able to do so has allowed a modicum of peace, of acceptance, and with that, a relative stillness, and a new starting point from which to fall upward into the second stage of my life (Rohr, 2013).

The more I have thought with Foucault over the years, the more I have realized that the effects of my docility have been far more reaching than I initially understood. I have come to understand that the residue of the disciplinary practices I experienced has remained and, like an undetected toxin, infiltrated numerous aspects of my existence. In one example, I now see quite clearly the connection between the effects of the disciplinary logic (Denison et al., 2017) that determined my football experiences and how I (oftentimes awkwardly) posture my meaning for exercise as a retired footballer (Jones and Denison, 2019).

Now, after the dust has settled, I continue to harness Foucault's notion of power as productive, as I try to exercise with greater nuance—rejecting exercise's former explicit purpose, that of controlling or improving my body for the purpose of performance. Rather, these days, when I pound frosty pavements that glitter like diamonds in the sun of early spring, or when I feel trickling sweat sting my eyes on hot summer nights, I am thirsting for an unadulterated appreciation of movement and rhythm. I take pleasure in striving to be a nomad of exercise, dislocated from any transformative goal. It is not easy. I still feel the tickle, the scratch of familiar corrective thoughts. But, as these thoughts emerge, I do my work, I hold them in check, and I keep on jogging. It may be true that I remain a long way from home, and perhaps I am still a little lost. Yet in my disordered state I feel free to traverse new, unknown paths as they unfold before me. I find myself faced with a liberating conundrum that because of Foucault, I no longer feel I am required to resolve.

### Author Two's Vignette–Spring 2021

When I think back to my footballing days and my time spent at the national football training center in France in the early 2000's, I mainly see bodies moving to the orderly rhythms of an exact and exacting sporting discipline. Tightly controlled and monitored training sessions organized around a fine-tuned yearly periodized plan, every minute of training accounted for as we stretched each day toward the same, seemingly always receding, horizon of perfection and achievement.

Never mind if, slowly but surely, the joy of movement gradually disappeared from our strides; if our choices and decision-making on the field became more timid, less creative, more predictable; if we started to look and play a lot more like a well-oiled machine than individuals with different qualities and characteristics; if some fell by the wayside, failing to deliver on the promise of performance and, this, despite embracing the disciplinary logic of high-performance sport almost as fervently as new age zealots.

Back then I was very much convinced that this was the best way, the only way in fact. Why would I have thought otherwise? After all, was my coach not an expert? Did the national training center not embrace cutting-edge player development practices underpinned by the latest scientific developments? Had we not rightly traded a traditional “warrior” discourse in favor of a modern “enlightened” scientific approach to player development? Was this not the pinnacle of French elite development football, of international repute?

It was not until being exposed to the critical thinking of Michel Foucault 10 years later during my Master's and subsequent PhD that I started to revisit these questions and, in doing so, my own “failed” footballing subjectivity—a difficult process which fortunately also led me to re-think my relationship to movement and exercise after retiring from high-performance football. Challenged by Foucault to think differently, I sought to move differently. I embraced climbing in its various declinations as a means to practice my newly found poststructuralist sensibilities, paying close attention to the rhythms of my moving body engaging with new mediums of rock, snow, and ice. Places of geography became places for presencing.

The transition was not easy. In truth the pull of discipline and disciplinary pleasures and rewards never completely faded away; they simply gradually receded to the background, like white noise. There was no silver bullet or magic formula. I was not “cured.”

I'll admit that it is easier these days. I have tools to push back. Years of thinking critically about “all” that high-performance sport does (Denison and Avner, 2011; Avner et al., 2019) have enabled a gradual “affective unhooking” (Coffey, 2019) from the pull of sporting discipline and the associated pressure to train, continuously push oneself, and measure one's progression linearly against some fixed metric.

While problematizing the disciplinary legacy of high-performance sport has created the necessary distance for me to start to explore different ways of moving and being, it is something I have had to nurture. For instance, I have deliberately sought out supportive friends and climbers who privilege our shared experience in the mountains above getting to the top of a peak or climbing increasingly difficult climbs. I have tried (and at times failed) to resist the pressure to “train for climbing,” focusing instead on how my body feels and approaching skill development and movement with a more playful curiosity than I did for most of my footballing days.

Of course, there is nothing “wrong” with training to push one's technical grade or tracking one's progress along the way, but I for one am grateful that moving and climbing have become much more to me.

### Author Three's Vignette–Winter 2022

The images one stores in their mind can have a powerful effect on the “doing of sport.” As a runner my mind was always stacked with images associated with times, results, and rankings. There was always a target ahead to hit or reach. Standards, norms, measures…expectations and comparisons: these are the memories I carry from “being a runner.”

It might be difficult to do, but I recognize now that targets can be ignored and time can remain unmeasured. Does this mean becoming ignorant? Does it mean turning resistant? Or can it lead to a more liberating rethinking of doing sport?

When a workout went poorly or I underperformed in a race, the effects were always so lasting. The crush of disappointment and failure weighed heavy: a load to bear that seemed my fault but in another less clear way not my fault at all. That was a confusion I never resolved.

After moving to a winter city for a new academic position in 2007, taking up cross-country skiing made total sense. With my endurance background I was sure I would take to it quickly. And I did.

On cross-country skis, as I move over the snow, what I see in my mind are the shapes I am forming and the patterns I am repeating. And with those shapes and patterns comes a rhythm I am trying to learn. From a biomechanics perspective it is the percentage of time spent in the various phases of a movement that produces its rhythm. And from an ecological dynamics perspective coordination is the ability to retain that rhythm at different speeds and as conditions change. This is the ballet of our muscle contractions—concentric, eccentric, isometric—that makes cross-country skiing such a joy for me, a degree of pleasure and satisfaction I rarely felt as a runner.

So how is it that biomechanics and ecological dynamics, two members of the sport science family that as a good sport sociologist I am supposed to love to hate, have helped me to transform my understanding of the doing of sport? I think this is where Foucault can come in, and how his broader theorizing can allow for thinking in ways that are not so bound by tradition and disciplinary silos. For example, with my new knowledge concerning the “rhythm of skiing” I have been able to shift my focus away from a concentration on “becoming fit” to take true pleasure from mastering a skill. That's not something I understood as a young runner all those years ago—how could I have, really? As a result, running largely became an act of discipline prescribed by the seeming unquestionable dominance of exercise physiology (Denison, 2019). But, oh my, how one's thinking can change. And change for the better, I must say.

Just this winter (2022) this is what I have been trying to achieve; this is what I have been repeating to myself in order to see it in my mind's eye. *Feel the transfer of weight from one ski to the other…press and sink into the glide…good…hold it longer…now rise up, see the changes in angles, the shortening and lengthening…the rotations…the distribution of forces through the snow*.

There you have it, my gallery of cross-country skiing images. A gallery that requires constant diligence to sustain. The push all around me to become more precise and exact, to regulate, compare, and measure my fitness and form, is immense. It's up against this pressure that I remind myself of what running became to me and who I became as a runner; it's up against this pressure that I thank Foucault for helping me to think differently now.

## Foucauldian Commentary

There are many reasons why an individual's orientation to exercise may change over time. A change in disposition, a key life event, injury, illness, experiencing a new culture, to name a few. For us, it was our exposure to social theory, and in particular the thoughts of Michel Foucault. We of course acknowledge that reading social theory is not a pre-requisite for one to experience exercise differently in their retirement from sport, however, it has helped us to do so. Taken together, these narrative vignettes have “shown,” not only the effects on each of us of having been a docile body through an immersion in a high-performance sports culture typified by the traditional and normalized disciplinary logic of sport (Denison et al., 2017), but also, how a Foucauldian outlook forged through our academic training has afforded us the opportunity to experiment with different ways of moving and exercising as we live our lives as retired high-performance athletes. As our narratives also show, this process has been anything *but* linear and straightforward. In fact, all of us continue to wrestle with the familiar pull, and to some extent comfort, of sporting discipline—albeit to varying degrees and intensities. As author two put it, there is no silver bullet or magic formula. The “undoing” of discipline is a process which has and continues to require attentiveness and diligence—particularly in light of the ever-increasing push to quantify, track, monitor, and evaluate one's “progress” against a whole set of arbitrary metrics and targets (Jones et al., 2022). Undoing for us, therefore, is less about “finding a cure for the effects of docility” than about forming new relations: new relations with knowledge and new relations with ourselves and others. We are firm that adopting this stance and highlighting how we have embraced it to show how we orient differently to exercise in our vignettes disrupts the logic that currently presides regarding how to cope with retirement experiences.

While we have previously highlighted athletes' docility as a “significant obstacle to developing alternative meanings for exercise in retirement” (Jones and Denison, 2019, p. 841), in this paper, by showcasing our own experiences through Foucault, we tentatively suggest that this obstacle, while unavoidable, is not insurmountable. What is more, one way that we have found useful in understanding our exercise experiences is to use Foucault's thoughts as a heuristic to explore and think about this complex phenomenon in a new way. More specifically, in line with Foucault's (2003) own rationale for his critique of the penal system that he outlined in *Discipline and Punish* Foucault (1995), our concern with the making of docile bodies challenges the dominant system of thought. A form of rationality that, for over a century, has supported the notion that sport's norms, truth games, and long-received rules, traditions, procedures, and practices, provide the most effective and rational way to develop athletes. As a result, the various techniques and instruments, and material practices, that coaches employ and that can be used to produce docile athletes need to be continually scrutinized by coach educators, coaching scholars, and sport scientists for all that they do (MacLean, 2021).

Thankfully, our training as Foucauldian scholars has provided us with tools to “push back” against sport's dominant disciplinary logic. Central to all of our re-orientations to movement and exercise, is the Foucauldian process of problematizing. For Foucault (2003, 2006), to problematize involves a “defamiliarization” or a “rendering of the familiar strange.” It is this initial process of distanciation and “affective unhooking” (Coffey, 2019), which has enabled all of us to begin to identify the longer-term social implications of having “lived in sport,” including the lasting and manifold pernicious effects of sporting discipline in our lives. And as a result of this ongoing critical work, we have been able to explore alternative—arguably less limiting—ways of relating to our sports retirement, in our bodies, our movement, and our exercise. It is in this way that problematization for us intersects with ethics and social justice.

Clearly, we were all bothered by sport's disciplinary legacy for various reasons. Author One has identified how this legacy has left him with an unshakeable feeling of judgment that has persistently interfered with his attempts to exercise and relate to his moving body in ways foreign to those that defined his football career. For Author Two, long after leaving a high-performance football context this legacy manifested in an estranged, hyper-controlling and unhealthy relationship with her body as she sought to push its limits further and further in pursuit of new, always receding, horizons of perfection. And for Author Three it was the perpetual drawing up of sides and the persistence of binaries in determining what counted as right, best, and correct and what didn't. In all of these cases, it is clear that trust becomes subordinated by control, insecurity, and fear. And under such conditions how can anyone be expected to thrive—an athlete in the prime of their life, a coach trying to facilitate an athlete's potential, a retired athlete in pursuit of lasting health and mobility? Accordingly, what must be seen as becoming central to performance in sport is how problematization provokes an ongoing consideration of effects, ethics, and relations so that new more effective practices have the possibility, the oxygen and flexibility, if you will, to emerge.

Importantly, our “reorientations” to movement and exercise have not only brought about new movement practices, they have also brought about new forms of movement pleasure. For Author one “the unadulterated appreciation of movement and rhythm dislocated from any transformative goal”, for Author Two “the rhythms of her moving body as it engages with new mediums of snow, rock and ice” and for Author Three it is the “ballet of muscle contractions in cross-country skiing.” As Pringle (2010) illustrated, movement pleasures (and displeasures) are not intrinsic; rather they are socially produced and effectively managed through various social arrangements. As he argued, understanding how individuals are “oriented” toward certain types of movement pleasures and not others through these same social arrangements becomes a critical task. Reciprocally, providing insights into how individuals attempt to *actively* orient themselves toward different types of movement and movement pleasures, as we have done in this paper, is, we argue, equally important.

We are aware that each retired athlete has their own relationship with their exercise in retirement and that this complex relationship is influenced by multiple factors as individual's dispositions evolve over time. For us, we “arrived” at these qualitatively different bodily practices and movement experiences and insights through an in-depth problematization of our respective high-performance sporting cultures thanks to our training as sociocultural scholars of sport. However, in keeping with Foucault's (1983) injunction to “create ourselves as a work of art”, we do not view these new movement practices as end points to reach or, worse, “to stay true to,” but rather as a reflection of our evolving movement experimentations as we endeavor to move our bodies more consciously and with more playful curiosity in our post-high-performance sporting lives.

## Conclusion

In this paper, we have sought to marry creative narrative writing with Foucauldian theorization to help shed light on an under-researched aspect of the sport retirement experience—namely how former high-performance athletes find or attempt to find new meaning in movement and exercise after the dust has settled on their high-performance sporting lives. In doing so, this paper not only underlines the problematic legacy of high-performance sport for retiring athletes' relationship to movement and exercise, but also champions an important shift in how to think about the experience of sports retirement using social theory, and Foucauldian theorization in particular. Namely, to shift the focus away from aspects of retirement that have been comprehensively covered using the same procedures and paradigmatic assumptions, toward a new, as yet under-utilized, heuristic for the study of sports retirement. A heuristic that in this instance, has helped us to begin to explore the various legacies of high-performance sporting participation for retired athletes' relationship to exercise, movement, and their bodies. We believe such a shift will provide retirement scholars with a means of analysis to examine and appreciate with greater depth, that retirement experiences remain deeply connected to the relations of power that typified athletes' career experiences. As such, adopting this new approach will encourage sports retirement scholars to re-imagine their starting point, to ask new research questions thereby allowing for different understandings of the complex experience of retirement from sport to emerge. Here, by focusing upon relationships to movement and exercise, we have submitted to the reader the possibilities associated with a more nuanced way of “thinking about sports retirement with Foucault.” We frame this as a way of thinking that presents an alternative logic and vocabulary with which to problematize, deconstruct, and then to re-ascribe the complex, multi-dimensional experiences associated with the cessation of a high-performance sports career.

In closing, through our efforts here we suggest that those who study the experiences of the retired athlete might, as we have tried to do, slow down, and take a breath. In place of the intense normative quest for progressive transformation, we suggest that perhaps energy could be better spent in two ways. First, by quietly reflecting upon how previously inhabited social arrangements and “the legacy of docility” may be having a bearing upon how, in the current moment, former athletes orient their present relationship with their moving body. Second, by attempting to understand how “thinking with Foucault” can help interrupt and re-imagine the assumptions and relationships they may currently hold, including how they experience their movement and exercise.

## Author Contributions

LJ: original idea. LJ, ZA, and JD: all other aspects were produced collaboratively. All authors contributed to the article and approved the submitted version.

## Funding

This study was funded by the Social Sciences and Humanities Research Council of Canada (ID: RES0048703).

## Conflict of Interest

The authors declare that the research was conducted in the absence of any commercial or financial relationships that could be construed as a potential conflict of interest.

## Publisher's Note

All claims expressed in this article are solely those of the authors and do not necessarily represent those of their affiliated organizations, or those of the publisher, the editors and the reviewers. Any product that may be evaluated in this article, or claim that may be made by its manufacturer, is not guaranteed or endorsed by the publisher.

## References

[B1] Agnew D. Drummond M. (2015). Always a footballer? The reconstruction of masculine identity following retirement from elite Australian football. Qual. Res. Sport Exer. Health 7, 68–87. 10.1080/2159676X.2014.888588

[B2] Anderson L. (2006). Analytic auto-ethnography. J. Contemp. Ethnogr. 35, 373–395.

[B3] Avner Z. Denison J. Markula P. (2019). “Good athletes have fun”: a Foucauldian reading of university coaches' use of fun. Sports Coach. Rev. 8, 43–61.

[B4] Barcza-Renner K. Shipherd A. Basevitch I. (2020). A qualitative examination of sport retirement in former NCAA division I athletes. J. Athl. Dev. Exp. 2, 1–13. 10.25035/jade.02.01.01

[B5] Barker-Ruchti N. Tinning R. (2010). Foucault in leotards: Corporeal discipline in women's artistic gymnastics. Sociol. Sport J. 27, 229–250. 10.1123/ssj.27.3.229

[B6] Barth M. Gullich A. Forstinger C. Schlesinger T. Schroder F. Emrich E. (2020). Retirement of professional soccer players – a systematic review from social science perspectives. J. Sports Sci. 39, 903–914. 10.1080/02640414.2020.1851449 33295256

[B7] Bloomer M. Hodkinson P. (2000). Learning Careers: continuity and change in young people's dispositions to learning. Br. Educ. Res. J. 26, 583–597.

[B8] Brewer B. Van Raalte J. Linder D. (1993). Athletic identity: Hercules' muscles or Achilles' heel? Int. J. Sport Psychol. 24, 237–254. 10.1037/t15488-000

[B9] Camiré M. Trudel P. Forneris T. (2012). Coaching and transferring life skills: Philosophies and strategies used by high school coaches. Sport Psychol. 26, 243–260.

[B10] Carless D. Douglas K. (2009). ‘We haven't got a seat on the bus for you' or ‘all the seats are mine': Narratives and career transition in golf. Qual. Res. Sport Exerc. 1, 51–66. 10.1080/19398440802567949

[B11] Carless D. Douglas K. (2011). Sport and Physical Activity for Mental Health. Hoboken, NJ: John Wiley & Sons.

[B12] Chang H. (2008). Autoethnography as Method. Walnut Creek, CA: Left Coast Press Inc.

[B13] Clark M. Markula P. (2017). Foucault at the *barre* and other surprises: a case study of discipline and docility in the ballet studio. Qual. Res. Sport Exer. Health 9, 435–452. 10.1080/2159676X.2017.1309451

[B14] Coakley J. (1992). Burnout amongst adolescent athletes: a personal failure or social problem? Sociol. Sport J. 9, 271–285. 10.1123/ssj.9.3.271

[B15] Coakley J. (2015). Assessing the sociology of sport: On cultural sensibilities and the great sport myth. Int. Rev. Soc. Sport 50, 4–5. 10.1177/1012690214538864

[B16] Coffey J. (2019). Creating distance from body issues: Exploring new materialist feminist possibilities for renegotiating gendered embodiment. Leis. Sci. 41, 72–90. 10.1080/01490400.2018.1539685

[B17] Crocket H. (2014). “I had no desire to be having this battle with this faceless man on the soccer field anymore”: exploring the ethics of sporting retirement. Soc. Sport J. 31, 185–201. 10.1123/ssj.2012-0109

[B18] Crocket H. (2015). Foucault, flying discs and calling fouls: Ascetic practices of the self in Ultimate Frisbee. Sociology of Sport Journal 32, 89–105. 10.1123/ssj.2013-0039

[B19] Curran C. (2015). Post-playing careers of Irish born footballers in England, 1945-2010. Sport Soc. 18, 1273–1286. 10.1080/17430437.2015.1042970

[B20] Cushion C. Jones R. (2006). Power, discourse, and symbolic violence in professional youth soccer: The case of Albion football club. Sociol. Sport J. 23, 142–161. 10.1123/ssj.23.2.142

[B21] Denison J. (2007). Social theory for coaches: a Foucauldian reading of one athlete's poor performance. Int. J. Sports Sci. Coach. 2, 369–383. 10.1260/174795407783359777

[B22] Denison J. (2016). Social theory and narrative research: A point of view. Sport Educ. Soc. 21, 7–10.

[B23] Denison J. (2019). What it really means to ‘think outside the box': why Foucault matters for coach development. Int. Sport Coach. J. 6, 354–358. 10.1123/iscj.2018-0068

[B24] Denison J. Avner Z. (2011). Positive coaching: ethical practices for athlete development. Quest 63, 209–227. 10.1080/00336297.2011.10483677

[B25] Denison J. Jones L. Mills J. (2019). Becoming a good enough coach. Sports Coach. Rev. 8, 1–6. 10.1080/21640629.2018.1435361

[B26] Denison J. Mills J. Konoval T. (2017). Sports' disciplinary legacy and the challenge of ‘coaching differently.’ Sport Educ. Soc. 22, 772–783. 10.1080/13573322.2015.1061986

[B27] Denison J. Mills J. Jones L. (2013). Effective coaching as a modernist formation: A Foucauldian critique, in The Routledge Handbook of Sports Coaching, eds Potrac P. Gilbert W. Denison J. (London: Routledge), 388–399.

[B28] Edwards M. Rowe K. (2019). Managing sport for health: An introduction to the special issue. Sport Manage. Rev. 22, 1–4.

[B29] Eime R. Young J. Harvey J. Payne W. (2013). Psychological and social benefits of sports participation: The development of health through sport conceptual model. J. Sci. Med. Sport 16, 59–83. 10.1016/j.jsams.2013.10.190

[B30] Ellis C. Bochner A. (2000). Autoethnography, personal narrative, reflexivity: Researcher as subject, in Handbook of Qualitative Research, 2nd edn, eds Denzin N. Lincoln Y. (London: Sage), 733–768.

[B31] Feddersen N. Morris R. Littlewood M. Richardson D. (2020). The emergence and perpetuation of a destructive culture in an elite sport in the United Kingdom. Sport Soc. 23, 1004–1022. 10.1080/17430437.2019.1680639

[B32] Foucault M. (1978). The History of Sexuality: An Introduction, volume I. New York: Vintage.

[B33] Foucault M. (1982). The subject and power. Crit. Inq. 8, 777–795. 10.1086/448181

[B34] Foucault M. (1983). Afterword by Michel Foucault, in Michel Foucault: Beyond structuralism and hermeneutics, eds Dreyfus H. L. Rabinow P. (Chicago, IL, USA: University of Chicago Press).

[B35] Foucault M. (1984). What is enlightenment? In Foucault Reader, eds Rabinow P. (New York: Pantheon).

[B36] Foucault M. (1995). Discipline and Punish: The Birth of a Prison. London, England: Penguin Books.

[B37] Foucault M. (2003). The Essential Foucault: Selections From Essential Works of Foucault (1954-1984), eds P. Rabinow Rose N. S. (New York, NY: New Press).

[B38] Foucault M. (2006). History of Madness. London: Routledge.

[B39] Fuller R. (2014). Transition experiences out of intercollegiate athletics: a meta-synthesis. Qual. Rep. 19, 1–15. 10.46743/2160-3715/2014.1131

[B40] Gearity B. T. Mills J. P. (2012). Discipline and punish in the weight room. Sports Coach. Rev. 1, 124–134. 10.1080/21640629.2012.746049

[B41] Gerdin G. Pringle R. Crocket H. (2019). Coaching and ethical self-creation: problematizing the “efficient tennis machine”. Sports Coach. Rev. 8, 25–42. 10.1080/21640629.2018.1429114

[B42] Heikkala J. (1993). Discipline and excel: technologies of self and body and the logic of competing. Sociol. Sport J. 10, 397–412. 10.1123/ssj.10.4.397

[B43] Hickey C. Kelly P. (2008). Preparing *not* to be a footballer: higher education and professional sport. Sport Educ. Soc. 4, 477–494. 10.1080/13573320802445132

[B44] Hickey C. Roderick M. (2017). The presentation of possible selves in everyday life: the management of identity among transitioning professional athletes. Sociol. Sport J. 34, 270–280. 10.1123/ssj.2017-0018

[B45] Hill P. Lowe B. (1974). The inevitable metathesis of the retiring athlete. Int. Rev. Sociol. of Sport 9, 5–32. 10.1177/101269027400900301

[B46] Holt N. Deal C. Pankow K. (2020). Positive youth development through sport, in Handbook of Sport Psychology, eds Tenenbaum G. Eklund R. (Hoboken, NJ: John Wiley & Sons).

[B47] Johns D. P. Johns J. S. (2000). Surveillance, subjectivism and technologies of power: an analysis of the discursive practice of high performance sport. Int. Rev. Sociol. Sport 35, 219–234. 10.1177/101269000035002006

[B48] Jones L. (2020). Reading John Grisham's ‘Bleachers' with Foucault: Lessons for sports retirement. Sports Coach. Rev. 9, 168–184. 10.1080/21640629.2019.1603042

[B49] Jones L. Denison J. (2017). Challenge and relief: a Foucauldian analysis of retirement from football. Int. Rev. Sociol. Sport 52, 924–939. 10.1177/1012690215625348

[B50] Jones L. Denison J. (2019). Jogging not running: a narrative approach to exploring ‘exercise as leisure' after a life in elite football. Leis. Stud. 38, 831–844. 10.1080/02614367.2019.1662831

[B51] Jones L. Konoval T. Toner J. (2022). Sport and Surveillance Technologies, In Sport, Social Media, and Digital Technology: Sociological approaches, eds Sanderson J. 10.1108/S1476-285420220000015020

[B52] Jowett S. Kerr G. MacPherson E. Stirling A (2020). Experiences of bullying victimization in female interuniversity athletics. Int. J. Sport Exer. Psychol. 18, 818–832. 10.1080/1612197X.2019.1611902

[B53] Konoval T. Denison J. Mills J. (2019). The cyclical relationship between physiology and discipline: One endurance running coach's experiences problematizing disciplinary practices. Sports Coach. Rev. 8, 124–148.

[B54] Lally P. (2007). Identity and athletic retirement: a prospective study. Psychol. Sport Exer. 8, 85–99. 10.1016/j.psychsport.2006.03.003

[B55] Lehman E. Hein M. Baron S. Gersi C. (2012). Neurodegenerative causes of death among retired National Football League players. Neurology 79, 1970–1974. 22955124 10.1212/WNL.0b013e31826daf50 PMC4098841

[B56] MacKay S. Dallaire C. (2013). Skirtboarders. com: Skateboarding women and self-formation as ethical subjects. Sociol. Sport J. 30, 173–196. 10.1123/ssj.30.2.173

[B57] MacLean J. (2021). ‘The lieutenants of coaching': How materiality shapes coach developers' practices. Somatechnics 11, 265–282. 10.3366/soma.2021.0355

[B58] Manley A. Williams S. (2022). ‘We're not run on numbers, we're people, we're emotional people': Exploring the experiences and lived consequences of emerging technologies, organizational surveillance and control among elite professionals. Organization 29, 1–22. 10.1177/1350508419890078

[B59] Markula P. Pringle R. (2006). Foucault, Sport, and Exercise: Power, Knowledge, and Transforming the Self. London, England: Routledge.

[B60] Martin L. A. Fogarty G. J. Albion M. J. (2014). Changes in athletic identity and life satisfaction of elite athletes as a function of retirement status. J. Appl. Sport Psychol. 26, 96–110. 10.1080/10413200.2013.798371

[B61] McMahon J. Penney D. Dinan-Thompson M. (2012). ‘Body practices—exposure and effect of a sporting culture?' Stories from three Australian swimmers. Sport Educ. Soc. 17, 181–206. 10.1080/13573322.2011.607949

[B62] Mihovilovic M. (1968). The status of former sportsmen. Int. Rev. Soc. Sport 3, 73–93. 10.1177/10126902680030010516086200

[B63] Mills J. Denison J. (2013). Coach Foucault: problematizing endurance running coaches' practices. Sports Coach. Rev. 2, 136–150.

[B64] Mills J. P. Denison J. (2018). How power moves: a Foucauldian analysis of (in)effective coaching. Int. Rev. Sociol. Sport 53, 296–312. 10.1177/1012690216654719

[B65] Park S. Lavallee D. Tod D. (2013). Athletes' career transition out of sport: a systematic review. Int. Rev. Sport Exer. Psychol. 6, 22–25. 10.1080/1750984X.2012.687053

[B66] Pringle R. (2010). Finding pleasure in physical education: a critical examination of the educative value of positive movement affects. Quest 62, 119–134. 10.1080/00336297.2010.10483637

[B67] Rail G. Harvey J. (1995). Body at work: Michel Foucault and the sociology of sport. Sociol. Sport J. 12, 164–179. 10.1123/ssj.12.2.164

[B68] Rinehart R. (2010). Poetic sensibilities, humanities, and wonder: toward an e/affective sociology of sport. Quest 62, 184–201.

[B69] Roderick M. (2006). The Work of Professional Football: A Labour of Love? London, UK: Routledge.

[B70] Roderick M. (2014). From identification to dis-identification: case studies of job loss in professional football. Qual. Res. Sport Exer. Health 6, 143–160. 10.1080/2159676X.2013.796491

[B71] Rohr R. (2013). Falling Upward: A Spirituality for the Second Half of Life. New York, SPCK Publishing.

[B72] Sanders G. Stevinson C. (2017). Associations between retirement reasons, chronic pain, athletic identity, and depressive symptoms among former professional footballers. Eur. J. Sport Sci. 17, 1311–1318. 10.1080/17461391.2017.1371795 28911275

[B73] Shogan D. (1999). The Making of High Performance Athletes: Discipline, Diversity, and Ethics. Toronto, ON: University of Toronto Press.

[B74] Simon J. Docherty C. (2014). Current health-related quality of life is lower in former Division I collegiate athletes than in non–collegiate Athletes. Am. J. Sports Med. 42, 423–429. 10.1177/0363546513510393 24318608

[B75] Stambulova N. Ryba T. Henriksen K. (2020). Career development and transitions of athletes: the International Society of Sport Psychology position stand revisited. Int. J. Sport Exer. Psychol. 10.1080/1612197X.2020.1737836

[B76] Stamp D. Potrac P. Nelson L. J. (2021). More than just a ‘Pro': a relational analysis of transition in professional football. Sport Educ. Soc. 26, 72–86. 10.1080/13573322.2019.1694503

[B77] Torregrosa M. Ramis Y. Pallares S. Azocar F. Selva C. (2015). Olympic athletes back to retirement: a qualitative longitudinal study. Psychol. Sport Exerc. 21, 50–56. 10.1016/j.psychsport.2015.03.003

[B78] Tracey J. Elcombe T. (2004). A lifetime of healthy meaningful movement: Have we forgotten the athletes? Quest 56, 241–260. 10.1080/00336297.2004.10491825

[B79] Tsang T. (2000). Let me tell you a story: a narrative exploration of identity in high-performance sport. Sociol. Sport J. 17, 44–59. 10.1123/ssj.17.1.44

[B80] Ungerleider S. (1997). Olympics athletes' transition from sport to workplace. Percept. Motor Skills 84, 1287–1295. 10.2466/pms.1997.84.3c.1287 9229448

[B81] van Ramele S. Haruhito A. Kerkhoffs G. Gouttebarge V. (2017). Mental health in retired professional football players: 12-month incidence, adverse life events and support. Psychol. Sport Exerc. 28, 85–90. 10.1016/j.psychsport.2016.10.009

[B82] Varghese M. Ruparell S. LaBella C. (2022). Youth athlete development models: a narrative review. Sport Health. 14, 20–29. 10.1177/19417381211055396 34758649 PMC8669922

[B83] Warriner K. Lavallee D. (2008). The retirement experiences of elite female gymnasts: Self-identity and the physical self. J. Appl. Sport Psychol. 20, 301–317. 10.1080/10413200801998564

